# The response of the soil microbial food web to extreme rainfall under different plant systems

**DOI:** 10.1038/srep37662

**Published:** 2016-11-22

**Authors:** Feng Sun, Kaiwen Pan, Akash Tariq, Lin Zhang, Xiaoming Sun, Zilong Li, Sizhong Wang, Qinli Xiong, Dagang Song, Olusanya Abiodun Olatunji

**Affiliations:** 1 CAS Key Laboratory of Mountain Ecological Restoration and Bioresource Utilization & Ecological Restoration Biodiversity Conservation Key Laboratory of Sichuan Province, Chengdu Institute of Biology, Chinese Academy of Sciences, Chengdu 610041, People’s Republic of China; 2 University of Chinese Academy of Sciences, Beijing, 100039, People’s Republic of China

## Abstract

An agroforestry experiment was conducted that involved four planting systems: monoculture of the focal species *Zanthoxylum bungeanum* and mixed cultures of *Z. bungeanum* and *Capsicum annuum, Z. bungeanum* and *Medicago sativa* and *Z. bungeanum* and *Glycine max*. Soil microbial food web (microorganisms and nematodes) was investigated under manipulated extreme rainfall in the four planting systems to assess whether presence of neighbor species alleviated the magnitude of extreme rainfall on nutrient uptake of the focal species by increasing the stability of soil food web. Our results indicate that in the focal species and *G. max* mixed culture, leaf nitrogen contents of the focal species were higher than in the monoculture and in the other mixed cultures under extreme rainfall. This result was mainly due to the significant increase under extreme rainfall of *G. max* species root biomass, resulting in enhanced microbial resistance and subsequent net nitrogen mineralization rate and leaf nitrogen uptake for the focal species. Differences in functional traits of neighbors had additive effects and led to a marked divergence of soil food-web resistance and nutrient uptake of the focal species. Climate change can indirectly alleviate focal species via its influence on their neighbors.

Anthropogenic disturbances are continuously causing climatic changes and challenges for ecosystem stability. Under the current scenarios of climate change, extreme precipitation is expected to increase over most regions of the world, particularly in the high-latitude and tropical regionsand^1^. In China, a significant increase in extreme precipitation has occurred in the west, in the mid–lower reaches of the Yangtze River, and in parts of the southwest^2^. The changes in precipitation regimes will be more spatially heterogeneous and less predictable than other climate changes and will leads to more intense flooding occur at local and regional scales^3^. Scientists have been committed to ecosystem properties to stabilize and increase community stability under climate change^4^,^5^.

Ecological stability consists of resistance and resilience^6^. Resistance is the degree of change caused by a disturbance. Resistance as an ecological concept draws the ecologist’s attention to how to increase the resistance and stability of a habitat to enable communities and species to overcome the effects of climate change. Various factors are involved in resistance mechanisms and ecological stability, such as plant community composition and soil food-web structure^7^.

As one property in an ecosystem, plant community composition can increase or decrease the community’s capacity for stability^8^,^9^,^10^,^11^. For example, previous studies suggested that neighbor communities had positive, negative or no effects on the performance of focal species under heavy rainfall^11^,^12^. Other studies suggested that legumes facilitate or compete with the focal species, depending on the legume species, under heavy rainfall^8^,^10^. Thus, the composition of shifting plant species is the main driver for the production of the focal species in response to extreme rainfall. However, scientific evidence for the response of both the ecosystem and the plants to extreme rainfall is still insufficient^13^. The degree to which the response of the focal species is determined by the response of its neighbors is poorly understood^14^. Moreover, most previous studies have focused on the relationship between the composition of the plant species and primary productivity. Few studies have examined the effects of the composition of plant species in increasing the uptake of nutrients for the focal species via soil food-web stability^15^. In theory, the presence of different plant species (especially the presence of legumes) with different above-and belowground functional traits could potentially increase soil food-web complexity and stability^16^,^17^. However, stronger competition among different species may also decrease the stability of the soil food web under extreme rainfall, even in the presence of a legume^10^. The present study examines these contradictory hypotheses by exploring the stability of soil microbial and nematode community responses to variations in plant community composition and extreme rainfall.

There is a close link between aboveground and belowground sub-systems because plants provide organic matter to decomposing microbes, which are an important part of the soil food web and provide nutrients to the soil by decomposing a plant’s dead organic matter^18^. Recent investigations showed that subordinate plant diversity increases nematode diversity, abundance and stability^17^. However, many researchers have revealed that the species identity has a stronger influence on the nematode community than the species richness^19^,^20^. Thus, it is not clear whether the positive biodiversity effect is due to species richness (true biodiversity effect) or to the individual species in a mixture (identical effect).

Extreme rainfall regimes will quickly change soil water content with consequences for habitat, soil food-web stability and resource availability, all of which may cascade onto plant growth^5^,^21^. Previous studies suggested that the soil microbial community stability indexes and the nematode stability indexes were not significantly affected by plant diversity against extreme rainfall disturbance, indicating a limited potential from plant diversity to buffer soil food webs under extreme rainfall^4^. However, Mariotte *et al*.^22^ suggested that subordinate species moderated drought effects on earthworm communities due to the better drought resistance of subordinate species. Taylor and Wolters found that oribatid mite communities in beech litter were less sensitive to summer drought than those in spruce litter due to the presence of drought-tolerant oribatid mite species, which were strongly related to the litter types^23^. Thus, the plant-soil system can buffer against drought events. Using soil microbial and nematode communities that also have strong species-specific relationships, we hypothesized that plant community composition might be important in affecting soil food-web stability and the nutrient uptake of the focal species under extreme rainfall.

An agroforestry system is an intercropped growth of forest trees and crop plants. This combination of plants helps to improve soil-available nitrogen, increase carbon sequestration, enhance soil biodiversity, reduce threats and ultimately increase agricultural production. Thus, an agroforestry system is one of the potential tools to mitigate climate change, especially under extreme climate conditions^24^.

*Zanthoxylum* is a widely growing genus in southwestern China, India, North America and Australia^25^. The fruits are used to make Sichuan pepper and have medicinal uses such as for toothache or rheumatism. However, *Zanthoxylum* species such as *Z. bungeanum* easily succumb under continuous heavy rainfall, resulting in decreased yield and quality of fruits and increasing mortality rate^26^. Most previous studies focused on increasing nutrient use in agroforestry systems; however, the effects of increasing soil food-web stability are neglected. These effects relate to the uptake of nutrients indirectly under extreme rainfall conditions.

The objective of this study was to assess whether an agroforestry system could increase the available nitrogen uptake of the focal species by stabilizing the soil microbial food web under extreme rainfall. The present study tested four hypotheses:1) specific species affect soil microbial and nematode communities, 2) extreme rainfall affects the soil food web differently under different plant communities, 3) legume species increase soil microbes and nematode stability, and 4) planting systems facilitate the focal species relay on species-specific neighbors under extreme rainfall.

## Results

### Soil properties

Two-way ANOVA showed that planting systems significantly (*P* < 0.05) affected soil NH_4_^+_^N, NO_3_^−^–N, dissolved organic carbon (DOC) and dissolved organic nitrogen (DON) (Table 1), extreme rainfall significantly (*P* < 0.01) affected soil water content, NH_4_^+_^N, NO_3_^−^-N and DOC (Table 1). There was a significant (*P* < 0.05) interaction between planting system and extreme rainfall on soil NO_3_^−^–N (Table 1).

In the normal rainfall treatment, the Z-M mixed culture significantly (*P* < 0.05) increased soil NH_4_^+_^N when compared with the monoculture and other mixed cultures (Table 1). Soil NO_3_^−^–N, DOC and DON were greater in the legume mixed culture (*P* < 0.05) plots than in the monoculture plots and the Z-C mixed culture. Under extreme rainfall, soil NH_4_^+_^N, DOC and DON were greater in the legume mixed culture (*P* < 0.05) plots than in the monoculture plots (Table 1).

### Microbial community

**Figure 1:**
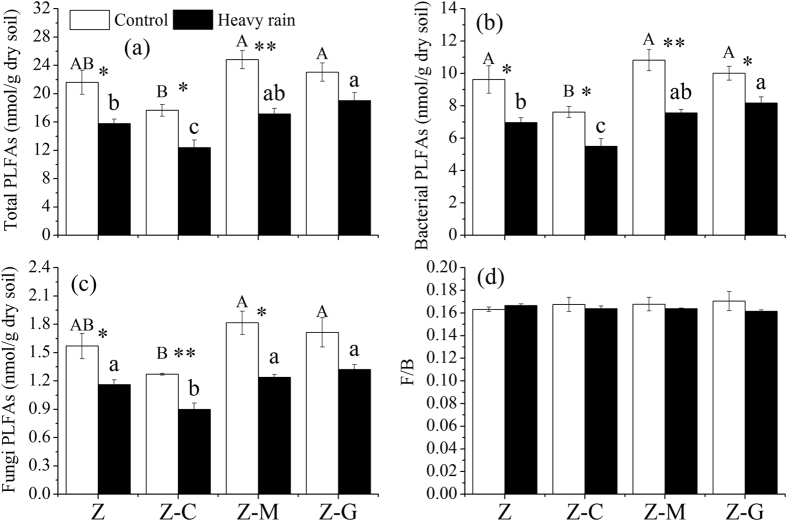
Response of microbial biomass in different planting systems under control and extreme rainfall. Mixed cultures of *Z. bungeanum* and *G. max* (Z-G), *Z. bungeanum* and *M. sativa* (Z-M), and *Z. bungeanum* and *C. annuum* (Z-C), and monoculture *Z. bungeanum* (Z). Different uppercase letters indicate significant differences among control (normal rainfall) treatments; different lowercase letters indicate significant differences among extreme rainfall treatments. Asterisks indicate significant differences between the bars (*P < 0.05, **P < 0.01; post hoc ANOVA).

The Z-M and the Z-G mixed cultures significantly (*P* < 0.05) increased total microbial, bacterial and fungal biomass compared with the Z-C mixed culture in normal rainfall condition (Fig. 1). However, the planting system did not have significant effects on the ratios of fungi to bacteria (Fig. 1d).

Compared with normal rainfall, extreme rainfall significantly (*P* < 0.05) decreased total microbial and fungal biomass in the Z, the Z-C and the Z-M cultures and significantly (*P* < 0.05) decreased bacterial biomass in the monoculture and mixed cultures (Fig. 1). Under extreme rainfall, the Z-G mixed culture significantly (*P* < 0.05) increased total microbial and bacteria biomass compared with the monoculture and the Z-C mixed culture. The Z-C mixed culture had a significantly (*P* < 0.05) lower fungal biomass than the monoculture and the legume mixed culture (Fig. 1c).

### Nematode community

**Figure 2:**
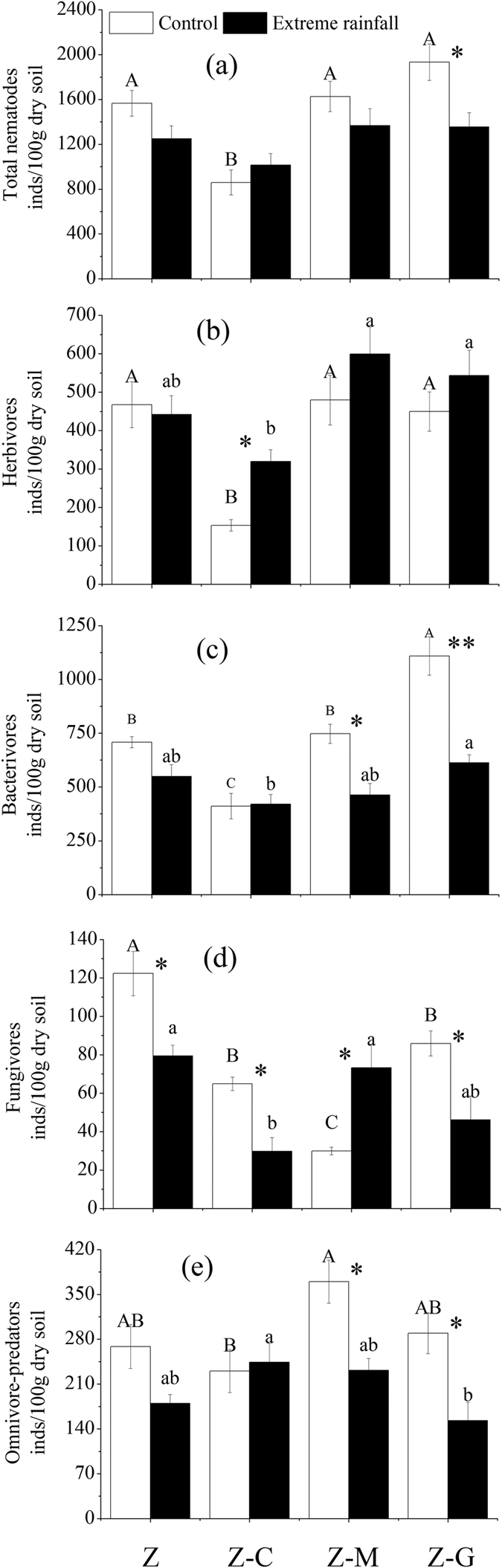
Response of nematode density in different planting systems under control and extreme rainfall. Mixed cultures of *Z. bungeanum* and *G. max* (Z-G), *Z. bungeanum* and *M. sativa* (Z-M), and *Z. bungeanum* and *C. annuum* (Z-C), and monoculture *Z. bungeanum* (Z). Different uppercase letters indicate significant differences among control (normal rainfall) treatments; different lowercase letters indicate significant differences among extreme rainfall treatments. Asterisks indicate significant differences between the bars (*P < 0.05, **P < 0.01; post hoc ANOVA).

Under normal rainfall, the Z-C mixed culture had significantly (*P* < 0.05) lower total nematode as well as herbivores and bacterivores densities than the monoculture and the legume mixed culture (Fig. 2). The Z-M mixed culture had significantly (*P* < 0.05) lower fungivores density than the monoculture and the Z-C and the Z-G mixed culture but had a significantly (*P* < 0.05) higher omnivore-predators density than the Z-C mixed culture (Fig. 2). The Z-G mixed culture had significantly (*P* < 0.05) greater bacterivores density than the monoculture and the other mixed cultures (Fig. 2c).

In the monoculture, extreme rainfall only significantly (*P* < 0.05) decreased fungivores density (Fig. 2d). In the Z-C mixed culture, extreme rainfall significantly (*P* < 0.05) increased herbivores density but significantly (*P* < 0.05) decreased fungivores density (Fig. 2b,d). In the Z-M mixed culture, extreme rainfall significantly (*P* < 0.05) decreased bacterivores and omnivore-predators density but significantly (*P* < 0.05) increased fungivores density (Fig. 2c–e). In the Z-G mixed culture, extreme rainfall significantly (*P* < 0.05) decreased total nematode as well as bacterivores, fungivores, and omnivore-predators density (Fig. 2).

The two-way ANOVA showed that planting systems significantly (*P* < 0.05) affected the density of *Pratylenchus, Rhabditis, Mesorhabditis, Acrobeloides, Alaimus, Aphelenchus, Ditylenchus,* and *Mesodorylaimus* (Table 2). Extreme rainfall significantly (*P* < 0.05) affected the density of *Pratylenchus, Rhabditis, Mesorhabditis, Aphelenchus, Ditylenchus,* and *Mesodorylaimus* (Table 2). The interaction effects of planting systems and extreme rainfall significantly (*P* < 0.05) affected the density of *Rhabditis, Mesorhabditis, Acrobeloides, Alaimus, Aphelenchus, Ditylenchus,* and *Mesodorylaimus* (Table 2).

Under normal rainfall, the monoculture significantly (*P* < 0.05) increased *Aphelenchus* density when compared to all of the mixed cultures. The Z-G mixed culture significantly (*P* < 0.05) increased the *Rhabditis* and the *Mesorhabditis* densities (Table 2).

### Resistance index

Under extreme rainfall stress, the Z-G mixed culture significantly (*P* < 0.05) increased the total microbe resistance index versus monoculture and the Z-C and the Z-M mixed culture but significantly (*P* < 0.05) decreased the bacterivores resistance index (Table 3). The Z-C mixed culture significantly (*P* < 0.05) decreased the herbivores resistance index compared with the monoculture, the Z-M and the Z-G mixed culture but significantly (*P* < 0.05) increased the omnivores-predators resistance index. The Z-M mixed culture had the lowest bacteria and fungivores resistance index (Table 3).

### Net nitrogen mineralization

**Figure 3:**
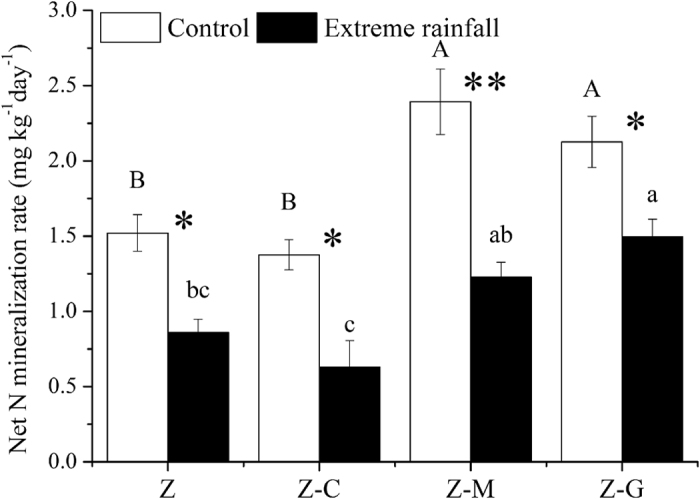
Response of net nitrogen mineralization rate in different planting systems under control and extreme rainfall. Mixed cultures of *Z. bungeanum* and *G. max* (Z-G), *Z. bungeanum* and *M. sativa* (Z-M), and *Z. bungeanum* and *C. annuum* (Z-C), and monoculture *Z. bungeanum* (Z). Different uppercase letters indicate significant differences among control (normal rainfall) treatments; different lowercase letters indicate significant differences among extreme rainfall treatments. Asterisks indicate significant differences between the bars (*P < 0.05, **P < 0.01; post hoc ANOVA).

Under normal rainfall, Z-M and Z-G significantly (*P* < 0.05) increased the net N mineralization rate when compared to Z and Z-C (Fig. 3). Extreme rainfall significantly (*P* < 0.05) decreased the net nitrogen mineralization rate in both the monoculture and the mixed culture. Under extreme rainfall, the Z-G mixed culture had a significantly (*P* < 0.05) higher net N mineralization rate than either the Z or the Z-C mixed cultures (Fig. 3).

### Plant leaf nitrogen

**Figure 4:**
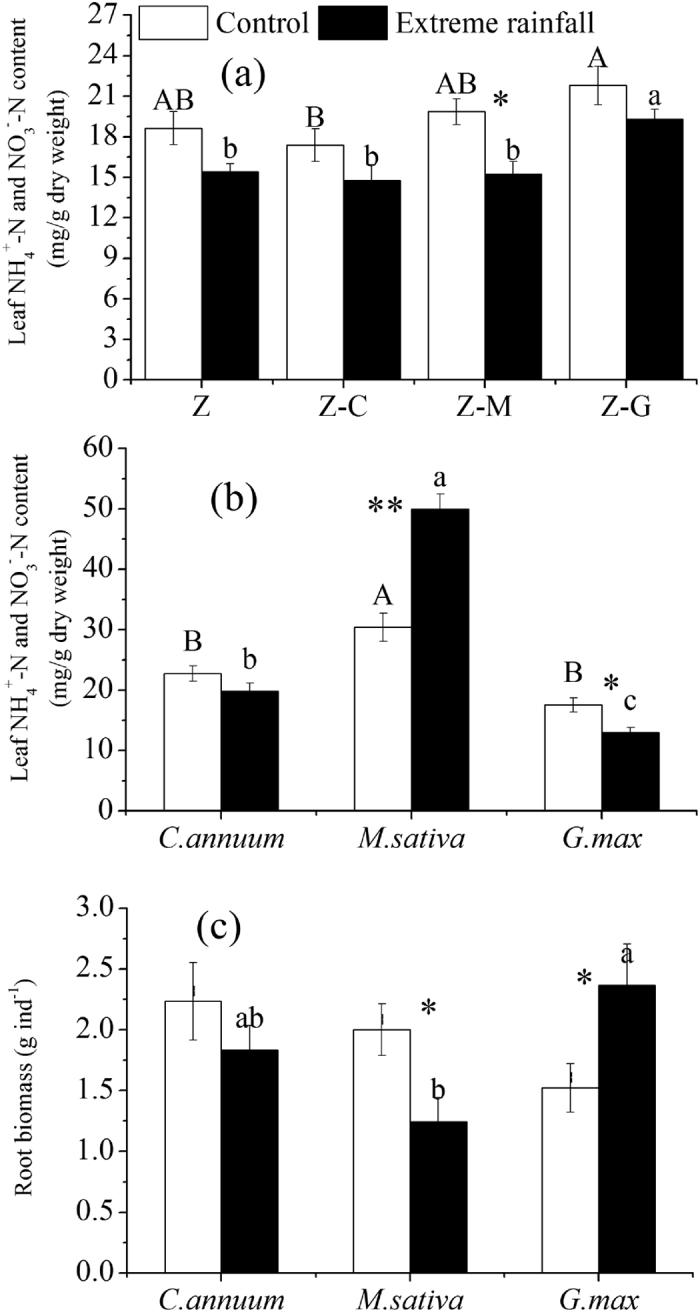
Response of leaf nitrogen contents in focal species and neighbor species and root biomass of neighbor species under control and extreme rainfall. Mixed cultures of *Z. bungeanum* and *G. max* (Z-G), *Z. bungeanum* and *M. sativa* (Z-M), and *Z. bungeanum* and *C. annuum* (Z-C), and monoculture *Z. bungeanum* (Z). Different uppercase letters indicate significant differences among control (normal rainfall) treatments; different lowercase letters indicate significant differences among extreme rainfall treatments. Asterisks indicate significant differences between the bars (*P < 0.05, **P < 0.01; post hoc ANOVA).

Under normal rainfall, the focal species of Z in the Z-G mixed culture had higher leaf nitrogen content than the Z-C mixed culture (*P* < 0.05) (Fig. 4a). The neighbors of M had significantly higher leaf N contents than C and G (Fig. 4b). Extreme rainfall significantly (*P* < 0.05) decreased Z leaf N contents in the Z-M mixed culture, extreme rainfall also tended to decrease Z leaf N contents in the monoculture (p = 0.077). However, extreme rainfall significantly (*P* < 0.05) increased M leaf N contents in the Z-M mixed culture. Under extreme rainfall, the focal species Z in the Z-G mixed culture had significantly (*P* < 0.05) higher leaf N contents than the monoculture, the Z-C or the Z-M mixed culture (Fig. 4a). The G species had the lowest leaf N contents when compared with C and M (Fig. 4b).

### Root biomass

Under normal rainfall, there was no significant difference between the neighboring species root biomass (Fig. 4c). Extreme rainfall significantly decreased the Z-M root biomass but significantly increased the Z-G root biomass. Under extreme rainfall, the Z-G mixed culture had a higher root biomass than the Z-M (Fig. 4c).

### Correlation analysis

Under extreme rainfall, the focal species of Z leaf N contents had a significant positive correlation with the total microbial resistance index, the bacterial resistance index and the net nitrogen mineralization rate but had a significant negative correlation with the total nematode resistance index, the bacterivores resistance index and the omnivores-predators resistance index (Table 4). However, the neighboring species leaf N contents had a significant negative correlation with the fungivores resistance index only (Table 4).

## Discussion

Legume addition significantly increased the soil-available N, DOC and DON, which was apparently attributed to the N-fixing ability of *G. max* and *M. sativa*. Therefore, the input of high-nitrogen residuals (litter and/or root exudates) to soils is the most likely reason for the enhancement of soil carbon and nitrogen content seen in this study. Furthermore, a previous study also recognized that the presence of legume species increased the soil carbon and nitrogen contents^27^. Extreme rainfall significantly decreased soil NO_3_^−^–N and DOC due to greater soil leaching.

Previous studies have found that mixed cultures (especially the presence of a legume) significantly increase microbial biomass because of enhanced litter and rhizodeposition input compared with monocultures^28^,^29^. However, in the present study, no significant differences were found in terms of total microbial, bacterial, fungal biomass and the ratio of fungi to bacteria in soils between the mixed cultures and the monoculture under normal rainfall (Fig. 1). This finding is possibly due to legume fixation of atmospheric N_2_ leading to higher levels of nitrogen in and around the plant (Table 1) and resulting in the rapid growth of bacteria. However, legume-induced changes in soil microorganisms might exert bottom-up control on higher trophic level organisms, such as microbivores and omnivore-predators, via the food web^28^. Our results also suggested that the legume mixed culture had higher bacterivores or omnivore-predator nematodes (Fig. 2). The legume mixed culture had a higher microbial biomass than the *C. annuum* mixed culture, likely because *C. annuum* produces poor-quality litter and root exudates compared to legume species^30^.

Previous studies have revealed that species identity, rather than species richness, has a stronger influence on nematode communities^19^,^20^. Given their ability to fixed N, legumes are the most common species that consistently influence soil nematodes^27^. Although a recent study argues that neighboring plant diversity increases nematode diversity, abundance and resistance^17^, the role of legumes as a key species in plant communities is well established. Likewise, our study provides evidence that legume addition significantly enhanced bacterivores in the *G. max* mixed culture and tended to increase omnivore-predator nematodes in the *M. sativa* mixed culture (p = 0.064) when compared to the monoculture (Fig. 2c,e). The *G. max* mixed culture significantly increased bacterivores mainly due to the significant increase of *Rhabditis* and *Mesorhabditis,* which both belong to *Ba1* bacterivores (Table. 2). *Ba1* bacterivores have short life spans and are known to rapidly enrich available resources^31^. NO_3_^−^–N, DOC and DON contents were higher in the *G. max* mixed culture soil, confirming its nutrient-rich status (Table 1). Therefore, nematode abundance maybe a useful measure to indicate available soil resources. Consistent with the present study, legumes supported large populations of certain bacterivores, especially *Rhabditis* and *Panagrolaimus*, both of which are *Ba1* nematodes^32^, and legumes increased bacterivores, most notably *r*-selected *Rhabditida*^19^. However, our results showed that another legume, the *M. sativa* mixed culture, remarkably increased *Alaimus (Ba4*) and *Mesodorylaimus (Op5*) (Table 2). Enhancements of some k-selected nematodes (especially Op5), which occupy high trophic levels in soil food webs, could increase the complexity of the soil food web^27^. The differences in the effects of the two legumes on soil nematodes might be due to plant chemistry. Indeed, plants supply food resources not only to plant-feeding nematodes but also to microbivores by forming litter above and belowground and by releasing large quantities of organic material from their living roots^33^. Different plant species vary greatly in terms of litter quality and root exudates; therefore, their decomposers are varied.

Previous studies had suggested that *C. annuum* could only inhibit root-knot herbivores by releasing different secondary metabolites^30^. The present study revealed interesting findings that *C. annuum* not only inhibits herbivores but also inhibits bacterivores and fungivores in the *C. annuum* mixed culture (Fig. 2c,d). Bacterial and fungal feeding nematodes play crucial roles in decomposing litter and increasing microbial activities in soil. Inhibitory effects on nematode density in the *C. annuum* mixed culture resulted in a significant decrease in the net nitrogen mineralization rate in comparison with the legume mixed culture (Fig. 3).

The *Z. bungeanum* monoculture had higher fungivores than the mixed cultures, mainly due to *Z. bungeanum’s* waxy leaf, which is difficult to decompose. Slow decomposition of poor-quality litter (low C/N) favors fungi and fungivores, while rapid decomposition of high-quality litter (high C/N) favors bacteria and bacterivores.

Taken together, our results suggest that the identities of a single plant species and plant functional groups are the most important factors influencing microbial and nematode communities. The above findings support our first hypothesis that specific species affect soil microbial and nematode communities.

Extreme rainfall regimes will increase soil water content rapidly with consequences for both soil food web and habitat^5^. However, very few studies have evaluated the effect of extreme rainfall on soil microbial communities^4^,^21^. The above two results suggest that extreme soil moisture significantly decreases microbial biomass and alters microbial communities because of low levels of oxygen and excessive amounts of water in the soil. These findings are consistent with our results that extreme rainfall significantly reduces total microbial, bacterial and fungal biomass (Fig. 1). First, an excessive amount of water in the soil causes the microbial cell membrane to rupture due to hypoxic condition^34^. Second, this marked decline in microbial biomass corresponds with a significant increase in nutrient loss in the soil (Table 1). This pattern suggests that extreme rainfall-induced leaching is likely to be linked to the decrease in microbial biomass. However, the F/B ratio did not show any significant change under extreme rainfall because of a simultaneous reduction in bacterial and fungal biomass (Fig. 1d). Therefore, this finding indicated that the fungi also did not have sufficient capacity to resist extreme soil water content stress^21^,^35^, although fungi have strong cell walls and intrinsic resistance potential against moisture stress events. This condition mainly exists because most fungi are located in/on the outer part of soil aggregates and are susceptible to extreme rainfall. Landesman and Dighton^36^ suggested no significant change in microbial biomass and community composition in response to extreme rainfall. This discrepancy may be due to different saturation levels and textures of tested soils. Landesman and Dighton^36^ investigated sandy soil with low water-holding capacity that cannot induce large fluctuations in soil moisture content, while in the present study, soil water content increased by up to 30%, and the soil had a higher capacity to hold water than sandy soil.

Nematodes respond rapidly to water stress because they depend upon water for movement and migration toward their prey in soil. Previous studies showed that increased soil moisture markedly decreased bacterivore abundance and increased herbivore density^37^,^38^. However, a recent investigation suggested that extreme rainfall stress increased total nematode abundance depending on the diversity of the plant species^4^. Our findings suggest that extreme rainfall had a significant effect on the density of bacterivores and herbivores depending on the composition of the plant species (Fig. 2b,c). Extreme rainfall significantly increased herbivore abundance in the *C. annuum* mixed culture (Fig. 2), possibly because high soil moisture content decreased the level of secondary metabolites released by *C. annum* to the soil. A higher abundance of herbivores in the soil could have serious negative effects on both soil and plants by feeding the roots of *C. annum* (Fig. 4c). Extreme rainfall significantly decreased bacterivores in the *G. max* mixed culture and *M. sativa* mixed culture (Fig. 2c). In the *G. max* mixed culture, a decrease in bacterivore abundance was mainly due to the decreased density of *Rhabditis* and *Mesorhabditis*, both of which belong to *Ba1* nematodes (Table 2). However, leaching induced by extreme rainfall had a limit effects on nematode dissemination^39^. The reason for this might be that extreme rainfall significantly increased *G. max’s* root biomass (Fig. 4c) and perhaps changed the root exudates, which negatively influenced *Ba1* nematodes in response to extreme rainfall^28^,^29^. Another reason might be that extreme rainfall disrupted the aggregate structure of soil where the habitable nematodes occur. In contrast to our study, a recent study suggested that *Ba1* nematodes increase after extreme rainfall because of their high acclimatization potential due to their high reproduction rate and impermeable cuticle^4^. However, that study showed nematode densities increasing strongly with increasing plant diversity after the extreme rainfall. Different plants have different strategies for withstanding flooding disturbances, e.g., by aerating the soil and thereby maintaining activity^40^. Thus, in the referenced study, a plant species’ existing tolerance to extreme rainfall in a community with high plant diversity was another reason for the increase in *Ba1* nematodes. In the *M. sativa* mixed culture in the present study, extreme rainfall mainly decreased the genera *Rhabditis, Acrobeloides* and *Alaimus* (Table 2). Nematodes responded differently in *G. max* and *M. sativa* mixed cultures under almost the same soil water content. Thus, the resistance of populations to soil organisms may be partly dependent on the traits of the respective plant species.

Extreme rainfall significantly decreased fungivores in monoculture as well as the *C. annuum* and *G. max* mixed cultures (Fig. 2). This pattern primarily occurred because extreme rainfall significantly decreased the genera *Aphelenchus* and *Ditylenchus*, which are cp-2 nematodes (Table 2). However, extreme rainfall significantly increased fungivores in the *M. sativa* mixed culture (Fig. 2). This result may be due to the significant reduction in omnivore-predator abundance. Thus, the food source or feeding relationship is also a dominant aspect for structuring the soil food web.

In the present study, extreme rainfall significantly decreased omnivore-predator abundance in *G. max* and *M. sativa* mixed cultures and tended to decrease them in the monoculture (p = 0.074). The density of the genus *Mesodorylaimus* (cp-5) decreased significantly in these cultures (Table 2). Cp-5 nematode species are the type most susceptible to environmental stress because of their long life cycle, permeable cuticle and high sensitivity. However, recent investigations revealed that Cp-5 nematode species have a strong resistance to different environmental stresses because of their varied and unidentified feeding strategies and their complex interactions with higher trophic levels^41^. All of the above results confirm our second hypothesis that extreme rainfall affected the soil food web differently under different planting systems.

Although a recent study argued that greater plant diversity is responsible for decreasing ecosystem stability under flooding stress^5^, ecosystem stability is directly related to plant functional diversity, e.g., nitrogen-fixing legumes^42^. Thus, no consistent conclusion exists about how plant community composition affects ecological stability, especially soil food-web resistance.

In the present study, total microbial resistance was higher in the *G. max* mixed culture than in the monoculture, the *C. annuum* mixed culture and the *M. sativa* mixed culture under extreme rainfall stress (Table 3). This finding was mainly due to the significantly increased root biomass of *G. max* under extreme rainfall, which greatly stimulated the growth of adventitious roots (F. Sun, personal observation) and increased tolerance to hypoxia, both of which safeguarded the root activity and functionality^43^. These factors also resulted in higher microbial resistance than was found in the monoculture or in the *C. annuum* and *M. sativa* mixed cultures. Another reason for this finding was that the reduction of bacterivores grazing on bacteria resulted in a higher microbial biomass than was found in the either the monoculture or the *C. annuum* mixed culture. Although the *M. sativa* mixed culture contained a legume species, the culture did not show an increase in microbial resistance (Table 3) because extreme rainfall significantly decreased the root biomass of *M. sativa* and reduced the input of root exudates to soil microorganisms. Therefore, microbial resistance depends on the composition and traits of the species. Bacterivores’ resistance was lower in the legume mixed culture than it was in the monoculture (Table 3) due to the stress extreme rainfall had on bacterivores and their abundance (Fig. 2). The composition of the species, even in the presence of the legume, could not enhance soil microbial and nematode resistance simultaneously. This apparent trade-off in the soil food web is used to constrain the composition and diversity of the entire soil community. Thus, these results only partly support our third hypothesis that legume species could increase soil microbes and nematode stability.

Neighbor plants have direct positive effects on whole plant communities by altering and modifying physical and/or biotic conditions^44^. Although the facilitation process is important for the structuring and functioning of an ecosystem, the mechanism behind this process is still poorly understood, especially in the belowground soil food web. Some studies suggest that leguminous plant presence enhances the N uptake of the focal species in ambient weather^45^ and in extreme rainfall^8^. However, our results were inconsistent with previous findings. Specifically, under extreme rainfall, only the *G. max* mixed culture had higher leaf nitrogen contents in the focal species when compared to the monoculture, the *C. annuum* mixed culture and the *M. sativa* mixed culture (Fig. 4a). The reason for this was that extreme rainfall significantly increased the *G. max* species root biomass, thereby enhancing microbial resistance and the net nitrogen mineralization rate. Thus, this process increased soil available N and enhanced nutrient uptake of the focal species. Our data also suggested that the focal leaf N contents had a significant positive correlation with total microbial resistance, bacterial resistance and the net nitrogen mineralization rate but a negative correlation with total nematode resistance and the bacterivore resistance index (Table 4). Thus, maintaining lower trophic level stability (microbial resistance) is more important than maintaining higher trophic level stability (nematode resistance) under extreme rainfall stress. In the *M. sativa* mixed culture, although a legume species was also present, the focal species had lower leaf nitrogen contents than the *G. max* mixed culture (Fig. 4a). The main reason was the strong interspecific competition for nitrogen between *M. sativa* and the focal species under extreme rainfall (Fig. 4b). The leaf nitrogen contents of *M. sativa* were significantly higher (+64%) than they were under normal rainfall. The deterioration effects for the focal species under extreme rainfall occurred because of increased competition for nutrients. These findings confirmed the study’s fourth hypothesis, that planting systems could facilitate the focal species’ relay on species-specific neighbor species under extreme rainfall. Legume species could facilitate the focal species, but the effect is highly species specific, related to climate change disturbance, and mostly due to belowground community processes. Thus, different mixed cultures would result in various soil food web structures under extreme rainfall and subsequently different biogeochemical cycling, soil function and nutrient availability. This finding is important in agricultural management that will continue to change to alleviate extreme climate conditions under future climate change. Belowground communities must be integrated into land management and climate change mitigation strategies.

## Materials and Methods

### Study site

The experiment was carried out in Maoxian, Sichuan Province, southwestern China (103°53′E, 31°41′N), at an altitude of 1686 m above the sea level. The mean annual temperature, precipitation and evaporation were 8.9 °C, 920 mm and 796 mm, respectively, according to meteorological monitoring from the Maoxian Ecological Station of Chinese Academy of Science. Most rainfall occurred in August, and most drought occurred in February. In this area, a significant increase in extreme precipitation was found in a previous study^2^. The soil was classified as Udic Luvisols^46^.

### Experimental design

Farmland soil with different organic fertilizers that is normally used to grow vegetables was use to establish the present experiment. The randomized complete block design (RCBD) was used to establish the experiment in April 2013. Four planting systems containing the monoculture *Zanthoxylum bungeanum* (Z), and the mixed cultures *Z. bungeanum* and *Capsicum annuum* L. (Z-C), *Z. bungeanum* and *Medicago sativa* L. (Z-M) and *Z. bungeanum* and *Glycine max* (L.) Merr. (Z-G) were intercropped in an agroforestry experiment. Two-year-old uniform seedlings of *Z. bungeanum* were collected from a local nursery based on plant stem base diameter and height. *Z. bungeanum* belongs to the family Rutaceae and is a perennial plant reproductively, while *M. sativa, G. max*, and *C. annuum* are annual plants. *M. sativa* and *G. max* are leguminous plant species, while *C. annuum* is an herbaceous plant. The focal species *Z. bungeanum* was grown once, in the center of each plot. The neighboring species *G. max, M. sativa* and *C. annuum* were planted at the same density in all plots. Each plot area was 2.6 × 2.6 m^2^, and the distance between each plot was 1 m. Each pattern was replicated six times randomly, for a total of 24 plots. In Z monoculture, all the understory plants were completely removed. All the planting systems had no fertilization from the establishment of experiment, and the understory grasses were removed by hand each week; herbicide was not used to avoid potentially negative effects on soil organisms^29^. In August 2015, we conducted precipitation treatments randomly. Control (normal rainfall) and extreme rainfall each had three replications. Control and extreme rainfall plots were maintained under rainout shelters during the experiment period. The shelters were constructed using steel frames and were covered with transparent plastic^47^ to protect from precipitation from August 1 to 30. To minimize greenhouse effects, the rainout shelters were 1.8 m aboveground. Tap water was used to mimic extreme precipitation events, and a watering pot was used to compensate for rain. Rain regimes were designated according to Ng *et al*.^48^ in which the average rainfall in the area during August was 3.0 mm/day (based on the average rainfall data during 1983–2013 from the Maoxian Ecological Station of Chinese Academy of Science), and was designated as the control rain regime, while extreme rainfall was designated according to the abnormally high rainfall in August (9.5 mm/day). All the plots were watered (7–9 am and 18–20 pm) from August 1 to 30. Around all plots, thick PVC panels were inserted at the top of the 0.5 m soil layer to prevent lateral water movement between plots and to prevent interaction between roots from neighboring plots.

### Field sampling

Soil sampling was conducted on 31 August 2015. A total of five soil cores (5 cm diameter) were collected using the five-spot method from a 10 cm soil depth and combined to form one composite sample for each plot. *Z. bungeanum* and neighbor species leaves were sampled on the same day, kept in a liquid nitrogen container, and taken back to the laboratory for determining the concentrations of leaf NH_4_^+^-N and NO_3_^−^-N. In total, three individual neighbor species were collected from each plot.

### Soil properties analysis

Estimation of soil moisture content was performed gravimetrically by oven drying (105 °C for 24 h) 20 g of the field soil sample. A button thermometer (QT-ST010, Beijing Channel Science Equipment Co., LTD, China) was used for soil temperature measurement, and data were recorded from each plot at 1-h time intervals. Soil pH was estimated in a 1:2.5 soil/CaCl_2_ (0.01 mol/L) suspension. Soil NH_4_^+^-N and NO_3_^−^-N were determined with the help of a flow injection auto analyzer (AA3, Bran+Luebbe, Germany). Dissolved organic carbon (DOC) and nitrogen (DON) were estimated using a TOC/TN analyzer (Multi N/C^®^2100(S), Analytik Jena AG, Germany). Net N mineralization rate was calculated according to the changes in NH_4_^+^-N and NO_3_^−^-N before and after the 7-d incubation.

### Phospholipid fatty acid (PLFA) analysis

Community structure and soil microbial biomass were determined using PLFA^49^. Eight grams of dry-weight-equivalent fresh soil was used for the extraction of lipids from a 23 mL extraction mixture containing chloroform:methanol:phosphate buffer (1:2:0.8 v/v/v). The concentration of each PLFA was calculated based on the 19:0 internal standard concentrations, and the abundance of the individual fatty acids was measured as nmol lipid per gram of dry soil. Total microbial biomass was assessed using total extracted PLFAs, with the sum of i15:0, a15:0, 15:0, i16:0, 16:1ω7, i17:0, a17:0, 17:0, cy17:0, 18:1ω7c and cy19:0 taken as an indicator of bacterial biomass, while 18:2ω6, 9c PLFAs were considered for fungi identification^28^,^50^. The above estimated biomass was used for the fungi to bacteria ratio.

### Nematode community analysis

The modified cotton-wool filter method was followed to determine soil (50 g) nematodes^51^. From each sample, the total number was identified, and then 100 nematode specimens were randomly selected and identified with the help of an inverted compound microscope. Nematode populations were expressed as the number of nematodes per 100 g dry soil. Based on their feeding habits, nematodes were categorized into four groups: (1) bacterivores, (2) fungivores, (3) omnivores-predators and (4) herbivores^52^.

### Resistance index analysis

Microbial and nematode resistance indexes were calculated according to Orwin and Wardle^6^, RS (t_0_) = 1−(2|D_0_|)/(c_0_ + |D_0_|), where c_0_ represents the control value after disturbance, while |D_0_| represents the difference between control and disturbed soil.

### Neighboring species root biomass analysis

All harvested neighboring root biomass was dried at 75 °C to constant weight and weighed.

### Plant leaf nitrogen analysis

Plants readily take up and directly use two main forms of soil available nitrogen (NH_4_^+^-N and NO_3_^−^-N) for growth. For leaf NH_4_^+^-N and NO_3_^−^-N measurement, 0.2-g leaf samples were homogenized in 2 mL of 10% HCL solution and 5 mL of deionized water, respectively. The resultant supernatants were analyzed using the quantitative colorimetric method^53^.

### Statistical analyses

Statistical analysis was performed using SPSS v. 17.0 (SPSS Inc., Chicago, IL). Two-way ANOVA was used to test the effects of planting systems and extreme rainfall on the soil properties and genera of nematode. One-way ANOVA was used to test the effects of planting systems on the soil properties, microbial biomass, nematode abundance, net nitrogen mineralization, microbial and nematode resistance index, neighbor species root biomass and leaf nitrogen content under control and extreme rainfall. One-way ANOVA was used to assess the effect of extreme rainfall on soil properties, microbial biomass, nematode abundance, net nitrogen mineralization, microbial and nematode resistance index, neighbor species root biomass and leaf nitrogen content under each planting system. Correlation analysis was used to assess soil biota resistance index and leaf NH_4_^+^-N and NO_3_^−^-N contents. Least significant difference (LSD) was used to test differences among treatment means. Statistical significance was determined at *P* < 0.05.

## Additional Information

**How to cite this article**: Sun, F. *et al*. The response of the soil microbial food web to extreme rainfall under different plant systems. *Sci. Rep.* **6**, 37662; doi: 10.1038/srep37662 (2016).

**Publisher’s note:** Springer Nature remains neutral with regard to jurisdictional claims in published maps and institutional affiliations.

## Figures and Tables

**Table 1:** Soil physical and chemical properties in study site.

Planting system	Z			Z-C			Z-M			Z-G			System	Rainfall	System* Rainfall
Soil properties	Control	Extreme rain	Sig.	Control	Extreme rain	Sig.	Control	Extreme rain	Sig.	Control	Extreme rain	Sig.			
Soil water content (%)	18.63 ± 0.80	30.26 ± 0.15	***	18.81 ± 0.24	30.72 ± 0.71	***	19.42 ± 0.67	31.82 ± 0.61	***	19.14 ± 0.54	30.46 ± 0.69	***	ns	***	ns
Soil temperature (°C)	15.59 ± 0.07	15.51 ± 0.10		15.42 ± 0.09	15.39 ± 0.03		15.90 ± 0.18	15.76 ± 0.06		16.10 ± 0.36	15.67 ± 0.07		ns	ns	ns
pH	7.62 ± 0.02	7.57 ± 0.01		7.67 ± 0.04	7.64 ± 0.03		7.59 ± 0.02	7.56 ± 0.03		7.57 ± 0.03	7.56 ± 0.01		ns	ns	ns
NH_4_^+_^N (mg/kg)	4.16 ± 0.41B	5.97 ± 0.66b		3.70 ± 0.14B	7.70 ± 0.61ab	**	6.07 ± 0.86 A	9.18 ± 0.99a		4.31 ± 0.49B	9.04 ± 0.94a	*	*	***	ns
NO_3_^−-^N (mg/kg)	17.81 ± 0.45B	9.62 ± 0.59	***	19.86 ± 1.05B	8.71 ± 0.64	**	24.94 ± 0.71 A	10.52 ± 1.01	***	25.19 ± 1.12 A	11.30 ± 0.79	**	***	***	*
DOC (mg/kg)	212.88 ± 1.06B	189.80 ± 4.43b	*	207.07 ± 1.70B	193.22 ± 3.63b	*	237.94 ± 7.07 A	223.27 ± 5.53a		229.37 ± 1.98 A	216.40 ± 4.11a	*	***	***	ns
DON (mg/kg)	55.25 ± 0.49B	52.82 ± 0.80b		56.14 ± 3.09B	53.61 ± 1.14b		64.89 ± 2.25 A	62.04 ± 2.85a		62.83 ± 0.89 A	61.43 ± 2.91a		***	ns	ns

Mixed cultures of *Z. bungeanum* and *G. max* (Z-G), *Z. bungeanum* and *M. sativa* (Z-M), and *Z. bungeanum* and *C. annuum* (Z-C), and monoculture *Z. bungeanum* (Z).

Different uppercase letters indicate significant differences among control (normal rainfall) treatments; different lowercase letters indicate significant differences among extreme rainfall treatments.

ANOVA was used to assess the effects of extreme rainfall on soil properties. *P < 0.05, **P < 0.01, ***P < 0.001.

**Table 2:** Response of nematode density in different plant patterns under control and extreme rainfall (Individuals per 100 g dry soil) (mean ± SE).

Planting system	Cp	Z			Z-C			Z-M			Z-G			System	Rainfall	System* Rainfall
Genus		Control	Extreme rain	Sig.	Control	Extreme rain	Sig.	Control	Extreme rain	Sig.	Control	Extreme rain	Sig.			
*Pratylenchus*	PP3	52 ± 15	114 ± 10ab	*	34 ± 7	87 ± 5bc	**	45 ± 15	48 ± 9c		56 ± 19	153 ± 29a	*	**	***	ns
*Rhabditis*	Ba1	167 ± 16B	108 ± 3a	*	115 ± 20B	36 ± 11b	*	122 ± 13B	75 ± 6a	*	563 ± 98 A	87 ± 17a	**	***	***	***
*Mesorhabditis*	Ba1	35 ± 12B	7 ± 4		0B	15 ± 4	*	25 ± 5B	11 ± 6		172 ± 19 A	7 ± 4	**	***	***	***
*Acrobeloides*	Ba2	99 ± 8 A	131 ± 25a		51 ± 2B	67 ± 8bc		92 ± 14 A	11 ± 6c	**	95 ± 16 A	89 ± 22ab		**	ns	**
*Alaimus*	Ba4	57 ± 8B	44 ± 3b		46 ± 2B	78 ± 6ab	**	130 ± 13 A	59 ± 13b	*	32 ± 3B	103 ± 15a	**	**	ns	***
*Aphelenchus*	Fu2	90 ± 12 A	45 ± 9	*	47 ± 1B	18 ± 6	*	8 ± 4 C	18 ± 6		27 ± 6BC	38 ± 11		***	*	**
*Ditylenchus*	Fu2	18 ± 5B	6 ± 3b		4 ± 2B	0 ± 0b		9 ± 5B	21 ± 4a		40 ± 11 A	4 ± 4a	*	*	*	**
*Diphtherophora*	Fu3	14 ± 2	10 ± 5ab		9 ± 3	6 ± 3b		9 ± 5	27 ± 9a		5 ± 5	4 ± 4b		ns	ns	ns
*Mesodorylaimus*	Op5	69 ± 16BC	10 ± 1c	*	26 ± 1 C	44 ± 10b		151 ± 26 A	66 ± 5a	*	123 ± 14AB	21 ± 5c	**	***	***	**

Mixed cultures of *Z. bungeanum* and *G. max* (Z-G), *Z. bungeanum* and *M. sativa* (Z-M), and *Z. bungeanum* and *C. annuum* (Z-C), and monoculture *Z. bungeanum* (Z).

Different uppercase letters indicate significant differences among control (normal rainfall) treatments; different lowercase letters indicate significant differences among extreme rainfall treatments.

ANOVA was used to assess the effects of extreme rainfall on soil properties. *P < 0.05, **P < 0.01, ***P < 0.001.

**Table 3:** Soil food web resistance index in different patterns under extreme rainfall.

Pattern	Z	Z-C	Z-M	Z-G
Resistance index				
Total microbes	0.58 ± 0.04b	0.54 ± 0.05b	0.53 ± 0.02b	0.71 ± 0.003a
Bacteria	0.58 ± 0.04ab	0.57 ± 0.04ab	0.55 ± 0.04b	0.69 ± 0.01a
Fungi	0.6 ± 0.04	0.55 ± 0.06	0.52 ± 0.03	0.64 ± 0.05
Total nematodes	0.67 ± 0.06	0.69 ± 0.04	0.75 ± 0.13	0.54 ± 0.03
Herbivores	0.91 ± 0.06a	−0.04 ± 0.02b	0.61 ± 0.16a	0.65 ± 0.16a
Bacterivores	0.63 ± 0.07b	0.87 ± 0.01a	0.46 ± 0.07c	0.38 ± 0.02c
Fungivores	0.48 ± 0.02a	0.3 ± 0.08a	−0.15 ± 0.12b	0.39 ± 0.13a
Omnivores-predators	0.53 ± 0.09b	0.87 ± 0.10a	0.46 ± 0.04b	0.38 ± 0.08b

Mixed cultures of *Z. bungeanum* and *G. max* (Z-G), *Z. bungeanum* and *M. sativa* (Z-M), and *Z. bungeanum* and *C. annuum* (Z-C), and monoculture *Z. bungeanum* (Z).

Different lowercase letters indicate significant differences among plant patterns under extreme rainfall treatments.

**Table 4:** Correlation analysis of soil biota resistance index and leaf NH_4_^+^-N and NO_3_^−^-N contents.

	Total microbe Resistance index	Bacteria Resistance index	Fungi Resistance index	Total nematodes Resistance index	Herbivores Resistance index	Bacterivores Resistance index	Fungivores Resistance index	Omnivore-predators Resistance index	Net Nitrogen mineralization rate
Z. bungeanum leaf N contents	0.786^**^	0.706^*^	0.404	−0.823^**^	0.329	−0.632^*^	0.245	−0.714^**^	0.667^*^
Neighboring species leaf N contents	−0.625	−0.565	−0.477	0.505	0.240	−0.211	−0.779^*^	−0.167	0.054

*P < 0.05, **P < 0.01.
